# Effects of Sleep Deprivation on Brain Bioenergetics, Sleep, and Cognitive Performance in Cocaine-Dependent Individuals

**DOI:** 10.1155/2013/947879

**Published:** 2013-10-22

**Authors:** George H. Trksak, Bethany K. Bracken, J. Eric Jensen, David T. Plante, David M. Penetar, Wendy L. Tartarini, Melissa A. Maywalt, Cynthia M. Dorsey, Perry F. Renshaw, Scott E. Lukas

**Affiliations:** ^1^Behavioral Psychopharmacology Research Lab, McLean Hospital, 115 Mill Street, Belmont, MA 02478, USA; ^2^McLean Imaging Center, McLean Hospital, 115 Mill Street, Belmont, MA 02478, USA; ^3^Sleep Research Laboratory, McLean Hospital, 115 Mill Street, Belmont, MA 02478, USA; ^4^Harvard Medical School, 25 Shattuck Street, Boston, MA 02115, USA; ^5^Charles River Analytics, Inc., 625 Mt. Auburn Street, Cambridge, MA 02138, USA; ^6^Department of Psychiatry, University of Wisconsin School of Medicine and Public Health, Madison, WI 53719, USA; ^7^Sleep Health Centers, 1505 Commonwealth Avenue, Brighton, MA 02135, USA; ^8^Private Practice, 1266 Main St., West Concord, MA 01742, USA; ^9^Department of Psychiatry, The Brain Institute, University of Utah School of Medicine, Salt Lake City, UT 84132, USA

## Abstract

In cocaine-dependent individuals, sleep is disturbed during cocaine use and abstinence, highlighting the importance of examining the behavioral and homeostatic response to acute sleep loss in these individuals. The current study was designed to identify a differential effect of sleep deprivation on brain bioenergetics, cognitive performance, and sleep between cocaine-dependent and healthy control participants. 14 healthy control and 8 cocaine-dependent participants experienced consecutive nights of baseline, total sleep deprivation, and recovery sleep in the research laboratory. Participants underwent ^[31]^P magnetic resonance spectroscopy (MRS) brain imaging, polysomnography, Continuous Performance Task, and Digit Symbol Substitution Task. Following recovery sleep, ^[31]^P MRS scans revealed that cocaine-dependent participants exhibited elevated global brain *β*-NTP (direct measure of adenosine triphosphate), *α*-NTP, and total NTP levels compared to those of healthy controls. Cocaine-dependent participants performed worse on the Continuous Performance Task and Digit Symbol Substitution Task at baseline compared to healthy control participants, but sleep deprivation did not worsen cognitive performance in either group. Enhancements of brain ATP levels in cocaine dependent participants following recovery sleep may reflect a greater impact of sleep deprivation on sleep homeostasis, which may highlight the importance of monitoring sleep during abstinence and the potential influence of sleep loss in drug relapse.

## 1. Introduction

Compared to healthy control individuals, cocaine-dependent individuals commonly exhibit disturbed sleep during cocaine use and abstinence [1]. Following cocaine use, rapid eye movement (REM) sleep decreases and slow wave sleep increases [1], and while sleep parameters appear similar to healthy control levels during early abstinence, sleep progressively deteriorates as abstinence progresses [2, 3]. During abstinence, cocaine-dependent men experience increased sleep onset latency and wake after sleep onset, reduced latency to REM, total sleep time, and sleep efficiency index, and almost no slow wave sleep [4]. Studies have shown that during early cocaine abstinence, subjective reports of daytime sleepiness increase, but during late abstinence when sleep architecture is at its worst, subjective sleepiness appears to improve [2]. Enhancements in sleep disturbances with prolonged cocaine abstinence and the effect this has on the likelihood of relapse to cocaine use indicate that monitoring or improving sleep quality should be a primary clinical focus as abstinence progresses [3]. 

A second problem facing cocaine dependent individuals as abstinence progresses is a worsening of performance on tasks assessing impulsivity (Iowa Gambling Task), immediate and delayed memory, and sustained attention [5, 6]. These deficits may be associated with sleep problems mentioned above, as decrements in procedural learning tend to worsen in parallel with worsening physiological measures of sleep quality [7]. Collectively, these data demonstrate that alterations in how cocaine-dependent individuals respond to sleep loss may be associated with a dysregulation of the homeostatic sleep processes. Sleep loss may have differential effects on cocaine dependent individuals in comparison with healthy control individuals. The identification of differential effects of sleep loss in cocaine-dependent individuals may be evident in measures of neural correlates, sleep physiology/behavior, and cognitive behavioral performance. Furthermore, these measurable changes may be highly relevant to the acquisition and perpetuation of chronic drug use and may have direct effects on relapse potential during abstinence.

Sleep deprivation studies have revealed a disruption in energy production and metabolism associated with sleep loss, such that extended wakefulness serves as an energetic challenge to the brain [8, 9]. In rodents, glycogen accumulated during sleep is mobilized during waking and decreases regionally during sleep deprivation [10]. Moreover, sleep deprivation results in altered expression of mRNA coding for regulators of glycogen synthesis and degradation [11, 12]. Although the effects on glycogen production are strain dependent, sleep deprivation disrupts glycogen production throughout the brain [13–15]. Beyond effects on glycogen, sleep deprivation and energy metabolism have been linked through adenosine triphosphate (ATP), a primary energy currency among cells. Notably, the nucleoside adenosine, primarily formed as a catabolic byproduct from ATP, is central to the function of the basal forebrain in the regulation of recovery sleep after sleep deprivation, which links, albeit indirectly, ATP metabolism and sleep homeostatic mechanisms [16]. 

^[31]^P MRS provides a noninvasive *in vivo* method to quantify the expression of high energy phosphates (*α*-, *β*-, and *γ*-nucleoside triphosphates; NTPs), which are vital for energy production in the brain. *β*-NTP is reflective of ATP, while *α*- and *γ*-NTP are less specific and include ATP, as well as adenosine di- and monophosphate. One study using ^[31]^P MRS found no change in high energy phosphates in the frontal lobe the morning after a night of total sleep deprivation [17]. However, a second study extended these results by also assessing brain high-energy phosphate metabolism after one night of recovery sleep. They also found no changes after the night of total sleep deprivation, but the morning after one recovery sleep night, total NTP, *γ*-NTP, *β*-NTP, and glycerophosphocholine (GPC; a measure of phospholipid degradation) were increased [18]. This study also found corresponding increases in morning and evening subjective sleepiness as well as the characteristic recovery sleep-associated increases in the perceived depth of sleep, slow wave sleep, and sleep efficiency index during the recovery sleep night [18]. A more recent study examined the effects of sleep deprivation in drug dependence on sleep and brain bioenergetics [19]. In particular, the study found in methadone-maintained opiate dependent subjects that recovery sleep did not contain the characteristic increase in sleep efficiency and that brain levels of ATP were increased both after sleep deprivation and following recovery sleep. 

The current study investigated effects of sleep deprivation in cocaine-dependent individuals, examining changes in brain bioenergetic metabolism, subjective and objective sleep, and assessments of cognitive performance over the course of one baseline night of sleep, one night of total sleep deprivation, and one recovery sleep night. ^[31]^P MRS brain imaging was used to assess the expression of brain bioenergetics with particular interest in NTP measures of ATP. Polysomnography assessed objective sleep, questionnaires assessed subjective sleep, and cognitive performance was assessed with the Digit Symbol Substitution Task and the Continuous Performance Task. These data were collected as a companion study to a study in methadone-maintained individuals [19]. Based on these previous findings in a drug-dependent population (methadone-maintained participants) and based upon findings in cocaine dependent individuals indicating variable changes in the occurrence of sleep abnormalities between baseline active use and the progression of abstinence, it was hypothesized that brain NTP levels would increase after total sleep deprivation in both groups, and that increases in NTP would be greater in cocaine-dependent participants; that cocaine-dependent participants would have disrupted sleep measurements following recovery sleep; that cocaine-dependent individuals would exhibit poorer performance on cognitive measures at baseline; and that sleep deprivation would result in greater performance decrements in cocaine dependent individuals compared to that of healthy controls.

## 2. Methods

### 2.1. Participants

Participants were 14 healthy control adults (50% female) and 8 nontreatment seeking cocaine-dependent adults (38% female). There were no significant differences between healthy control and cocaine dependent groups on sex (Table 1), but cocaine-dependent participants were older (*P* < 0.05). Participants were recruited via newspaper, radio, or online advertisements. After initial phone screening, participants visited the Sleep Research Laboratory at McLean Hospital. Participants gave informed consent, underwent a physical examination (electrocardiogram, complete blood panel including a test of liver function), drug urine screen (QuickTox Drug Screen Dipcard, Branan Medical Corporation, Irvine, CA), and pregnancy test (Stanbio QuPID, Studio Laboratory, Boerne, Texas). On all study nights, participants were again tested for drugs and pregnancy. Participants were free of any current Axis I disorders (Structured Clinical Interview for DSM-IV Disorders) [20], with the exception of cocaine dependence for cocaine dependent participants.

### 2.2. Sleep Deprivation Procedures

 Participants took part in a separate initial screening night of sleep with polysomnography and full respiratory monitoring (respiratory flow, effort, and oximetry) in order to rule out all primary sleep disorders (including sleep apnea) and to allow the participants to acclimate to sleeping in the sleep research center. Participants then underwent three separate consecutive study nights in the laboratory consisting of one baseline sleep night (8 hour time in bed), one night of total sleep deprivation (sleep deprivation: participants were awake for ≥36 consecutive hours), and one recovery night (recovery sleep: 8 hour time in bed). To ensure total wakefulness during all study nights and during all active sleep deprivation periods, participants remained in a natural settings laboratory (a home living room laboratory environment) or the sleep laboratory bedroom, where they were continuously monitored throughout experimentation by laboratory research technicians. 

### 2.3. Polysomnographic Recording

Electroencephalogram (EEG), electrooculogram, electromyogram, and electrocardiograph measurements were acquired using an Alice 4 Sleepware system (Philips Respironics, Andover, MA). Electrode placement was in accordance with standard polysomnography and scored by standard scoring criteria [21]. Objective sleep measures were defined as follows: wakefulness after sleep onset—the amount of awake after sleep onset, sleep efficiency index—the total time asleep as a proportion of the total time in bed, total sleep time—total minutes of sleep after sleep onset, slow wave sleep—the total minutes of slow wave sleep, and total time spent in rapid eye movement (REM) sleep, in minutes.

### 2.4. Cognitive Behavioral Testing

The Continuous Performance Task [22] and the Digit Symbol Substitution Task [23] were administered each study morning, and the Digit Symbol Substitution Task was also administered prior to each bedtime. To assess mood, participants were administered the Profile of Mood States [24]; to assess subjective sleepiness, participants were administered the Stanford Sleepiness Scale [25] and a visual analog scale (anchors-sleepy, alert) in the morning each study day and prior to bedtime.

### 2.5. Brain Magnetic Resonance Spectroscopy Imaging

 MRS imaging was performed on a 4Tesla whole-body (Varian/Unity-INOVA; Varian, Palo Alto, CA, USA) MR scanner operating at 170.31 MHz for proton measurement and 68.95 MHz for phosphorus measurement. A dual-frequency, transverse electromagnetic design volume head coil tuned to both proton and phosphorus frequencies was used for all imaging and spectroscopy experiments (Bioengineering Inc., Minneapolis, MN, USA).

### 2.6. Proton MRS

High-contrast, T1-weighted sagittal images of the entire brain were first acquired using a 3D magnetization-prepared FLASH imaging sequence allowing for clear differentiation between grey and white matter, clearly delineating between the different anatomical regions of interest. Acquisition parameters for the sagittal images were TE/TR = 6.2/11.4 ms, field-of-view (FOV) = 24 cm × 24 cm, readout duration = 4 ms, receive bandwidth = ±32 kHz, in plane matrix size = 256 × 256, in plane resolution = 0.94 mm, readout points = 512, axial plane matrix size = 16, axial plane resolution = 2.5 mm sagittal, and scan time = 1 minute, 15 seconds. High-resolution T1 weighted images were acquired in the transverse plane with the same imaging sequence, but 32 slices were collected of 4 mm nominal thickness, for a scan time of 2 minutes, 30 seconds.

### 2.7. Phosphorus MRS

**Figure 1:**
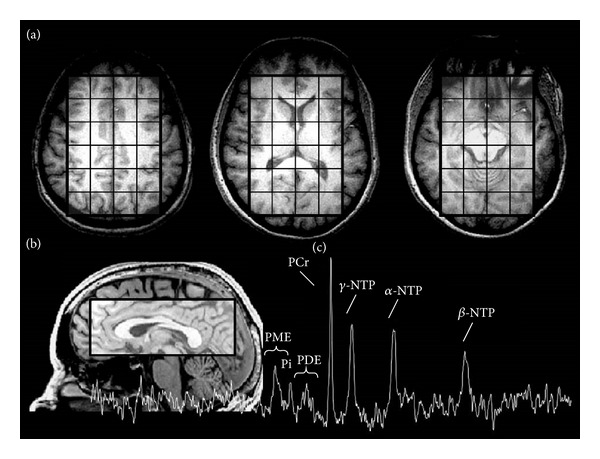
(a) Three representative axial brain slices illustrating voxel coverage for global whole brain  ^[31]^P MRS assessment, (b) illustrating a sagittal view of the voxelization of subcortical areas, and (c) a resultant  ^[31]^P MRS imaging spectra indicating phosphorus-containing peaks.

Phosphorus MRS was performed with the phosphorus channel of the dual-tuned proton-phosphorus head coil. Initially, eight control participants were scanned using a 3D ^[31]^P MRS sequence. It was recognized that the drug dependent participants would be either unable to complete the required >1-h scan duration of the 3D ^[31]^P MRS scan due to anxiety or excessive physical movement. To collect valuable scan data and to maintain comparability of scan data, a two-dimensional (2D) ^[31]^P MRS sequence was used for the remaining fourteen participants. Limiting the acquisition from a 2D slab allowed for a scan duration of 9 min compared to the 46-min 3D sequence. The 2D-MRSI sequence was phase encoded over a 6 cm thick excitation slab placed in the exact same mid-sagittal position as the 3 slices involved in the 3D sequence. All other parameters were identical between the 2D- and 3D-^[31]^P MRS protocols, including FOV (33 cm × 33 cm), TR (500 ms), matrix (16 × 16, sparse sampling scheme using the same SINC-lobe-modulated, weighted-average k-space filter). Transmit/receive frequency was first centered on the phosphocreatine (PCr) resonance, as measured with a global free-induction decay (FID). The 2D-CSI sequence used a reduced phase-encoding scheme [26], which allows for inclusion of circularly bound, reduced-point, weighted k-space acquisition, providing approximately 35% more signal-to noise for a given scan time-and effective voxel volume over conventional methods. All viable voxels from three mid-sagittal slices across the entire brain were analyzed from 3D ^[31]^P MRS data, which was essentially equivalent to the 2D axial-plane consisting of 2 cm × 2 cm × 6 cm voxels (slices are effectively 2 cm thick × 3 slices equal to 6 cm thick slab of interest). 2D-PSF (actual voxel signal distribution) and signal to noise were analogous between 2D and 3D ^[31]^P MRS acquisitions, and since the tip angle (32°) and TR were the same between sequences, the resultant spectra were virtually identical between sequences when tested back to back on a healthy control subject in terms of metabolite T1-weighting. Fundamental differences exist between the 2D-and 3D- ^[31]^P MRS sequences that primarily emanate from diminutive differences in the tip angle between sequences (global square pulse for 3D versus selective SINC pulse for 2D), leading to minute T1-weighted differences in derived peak areas. There is an inherent chemical-shift displacement artifact using 2D  ^[31]^P MRS, which could affect measures of any off-resonance metabolites due to spatial shifts in slab excitation. To rectify any potential influence, correction-factors for each measured metabolite from a healthy control participant were derived and for the 3D  ^[31]^P MRS data. The resultant metabolite measures were extremely in line with the acquired 2D  ^[31]^P MRS data, and there were no significant differences for any of the MRS metabolites. All *in vivo* CSI/image data were processed using Varian Nuclear Magnetic Resonance software, Version 6.1b (Varian, Palo Alto, CA, USA), and software developed on site. Prior to Fast Fourier Transform reconstruction to spatially resolve the CSI spectra, the collected k-space data was centered in a 16 × 16 square matrix, and each time-domain FID was zero filled out to 2048 complex points and left-shifted five points removing residual bone/rigid membrane signal. From the MRI images, the 2D-CSI data grid was shifted in the *x* and *y* dimensions to position the sampling grid, centered inside the brain according to anatomical landmarks. The peak areas of the metabolites: phosphoethanolamine (Pe), phosphocholine (PCh), inorganic phosphate (Pi), glycerophosphoethanolamine (GPE) and glycerophosphocholine (GPC), PCr, and three peaks for adenosine triphosphate (*α*-, *γ*-, and *β*-NTP; Figure 1) were fitted to an *in vivo* spectral model using a nonlinear, iterative [27, 28]. The fitting routine was based on a Marquardt-Levenberg algorithm, utilizing prior spectral knowledge for the relative amplitudes, linewidths, lineshapes, peak positions, and J-coupling constants to model the *in vivo* ^[31]^P brain spectrum. The fitted metabolite amplitudes were not T2-weighted since the fitting algorithm backextrapolates to time zero. Fitted spectral peak areas were expressed as a ratio to the total  ^[31]^P signal per voxel.

### 2.8. Statistical Analysis

Demographic characteristics were analyzed by *t*-tests. All other dependent measures were tested using linear mixed model ANOVAs with sleep night and treatment group as fixed factors. Because cocaine-dependent participants were older than healthy controls (*P* = 0.02), age was included as a covariate. Alpha was set to *P* < 0.05 (two-tailed) for all statistical tests and Bonferroni corrections were used for multiple comparisons.

## 3. Results

### 3.1. Magnetic Resonance Spectroscopy

**Figure 2:**
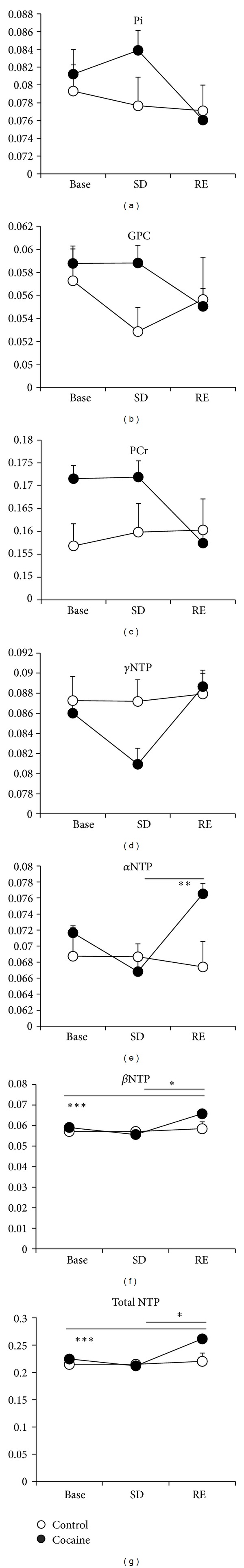
^[31]^P MRS global brain expression of inorganic phosphate (Pi; (a)), glycerophosphocholine (GPC; (b)), phosphocreatine (PCr; (c)), *γ*-nucleoside triphosphate (*γ*-NTP; (d)), *α*-NTP (e), *β*-NTP (f), and total NTP (g) in healthy control (control) and cocaine-dependent (cocaine) participants on the mornings of baseline (base; 8 hour time on bed), actively prolonged wakeful state following sleep deprived (SD), and recovery (RE; 8 hour time on bed) sleep. Significance (Sig.): *sleep night *P* < 0.05; **sleep night *P* < 0.01; ***sleep night *P* < 0.001.

 At baseline, there were no differences between cocaine-dependent participants and healthy controls for any metabolite. Importantly, there was a sleep night*group interaction for *α*-NTP (Figure 2(e); *F*_[2,59]_ = 4.02; *P* = 0.023), *β*-NTP (Figure 2(f), *F*_[2,59]_ = 3.95; *P* = 0.024), and total NTP (Figure 2(g); *F*_[2,59]_ = 3.59; *P* = 0.035). Cocaine-dependent participants had higher *α*-NTP after recovery sleep than after sleep deprivation (*P* = 0.008), higher *β*-NTP after recovery sleep than both baseline (*P* = 0.016) and sleep deprivation (*P* = 0.001), and higher total NTP after recovery sleep than both baseline (*P* = 0.043) and sleep deprivation (*P* = 0.001). There was no significant difference across the sleep deprivation paradigm for any metabolites among healthy controls. There was an effect of age on PCr (*F*_[1,59]_ = 4.63; *P* = 0.036); however, there were no significant effects of age for any NTP metabolite or GPC. 

### 3.2. Polysomnography

 For all polysomnography data, see Table 2. There were no baseline differences, no effect of age, and no sleep night*group interaction for any polysomnographic variable. In both groups on recovery sleep night, SOL was shorter (*F*_[1,39]_ = 4.75; *P* = 0.036), wakefulness after sleep onset was lower (*F*_[1,39]_ = 6.5; *P* = 0.015), sleep efficiency index was higher (*F*_[1,39]_ = 12.186; *P* = 0.001), total sleep time was longer (*F*_[1,39]_ = 14.0; *P* = 0.001), Stage 2 was longer (*F*_[1,37]_ = 4.54; *P* = 0.04), and REM was longer (*F*_[1,37]_ = 16.96; *P* < 0.0001). Across both sleep nights, cocaine dependent participants had fewer arousals compared to healthy control participants (*F*_[1,39]_ = 4.65; *P* = 0.037). 

### 3.3. Cognitive Performance

**Figure 3:**
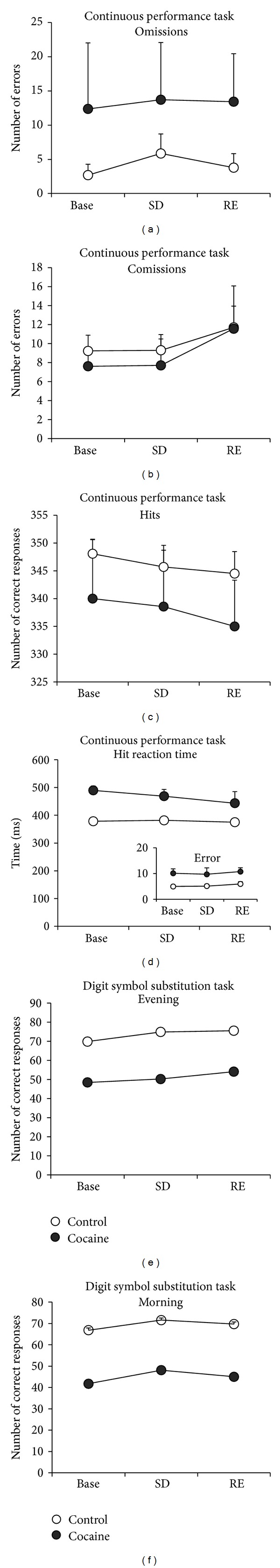
Differences between healthy control (control) and cocaine-dependent (cocaine) participants on Continuous Performance Task and for the Digit Symbol Substitution Task. The number of errors of omission (a) number, errors of commission (b), and the number of correct responses (hits; (c)), the hit reaction time (RT; (d)) hit RT standard error ((d) inset) on the CPT. The number of correct substitutions on the DSST as measured in the evening (e) and morning (f) of baseline (base; 8 hour time on bed), actively prolonged wakeful state following sleep deprived (SD), and recovery (RE; 8 hour time on bed) sleep.

At baseline, cocaine-dependent participants had slower reaction times (RTs) on correct responses (hits) on the Continuous Performance Task (Figure 3(d); *F*_[1,18]_ = 34.64; *P* < 0.001) and completed fewer accurate substitutions on evening (Figure 3(e); *F*_[1,29]_ = 4.63; *P* = 0.045), and morning (Figure 3(f); *F*_[1,18]_ = 6.82; *P* = 0.018) Digit Symbol Substitution Task than healthy controls. There was no effect of sleep night and no sleep night*group interaction, but across all four days of assessment, cocaine-dependent participants made more errors of omission (Figure 3(a); *F*_[1,56]_ = 4.46; *P* = 0.039), had slower hit RTs on the Continuous Performance Task (Figure 3(d); *F*_[1,56]_ = 25.25; *P* < 0.0001), and completed fewer accurate substitutions on the evening (Figure 3(e); *F*_[1,59]_ = 16.74; *P* < 0.0001) and morning Digit Symbol Substitution Task (Figure 3(f); *F*_[1,56]_ = 19.57; *P* < 0.0001). There was an effect of age on the evening Digit Symbol Substitution Task (*F*_[1,59]_ = 5.047; *P* = 0.028).

### 3.4. Subjective Sleep and Mood

 For all subjective sleep and mood data see Table 3. At baseline, cocaine-dependent participants reported lower sleepiness on the evening visual analog scale (*F*_[1,19]_ = 10.79; *P* = 0.004), higher restlessness (*F*_[1,17]_ = 7.43; *P* = 0.014), lower evening mood (*F*_[1,17]_ = 12.73; *P* = 0.002), more difficulty waking (*F*_[1,16]_ = 9.87; *P* = 0.006), and lower physical ailments (*F*_[1,18]_ = 8.34; *P* = 0.01). There was also an effect of age on ratings of restless (*F*_[1,17]_ = 7.94; *P* = 0.012) regardless of treatment condition.

All participants reported less vigor in the evening prior to recovery sleep than baseline nights (*F*_[2,56]_ = 5.05; *P* = 0.01), more fatigue in the evening prior to recovery sleep than baseline (*F*_[2,56]_ = 22.73; *P* < 0.0001) and sleep deprivation (*P* < 0.0001), more fatigue on morning ratings after sleep deprivation than baseline nights (*F*_[2,56]_ = 7.94; *P* = 0.001); and after sleep deprivation than recovery sleep nights (*P* = 0.024) on the Profile of Mood States. All participants also reported being more tired on the evening Stanford Sleepiness Scale prior to recovery sleep than baseline nights (*F*_[2,55]_ = 15.76; *P* < 0.0001) and prior to recovery sleep than sleep deprivation nights (*P* < 0.0001). Finally, all participants reported being sleepier on the evening visual analog scale after sleep deprivation than baseline nights (*F*_[2,58]_ = 16.31; *P* = 0.015) after recovery sleep than baseline nights (*P* = 0.02) and after recovery sleep than sleep deprivation nights (*P* < 0.0001). 

Across all sleep nights, cocaine-dependent participants reported greater depression on evening ratings (*F*_[1,56]_ = 11.11; *P* = 0.002), less evening vigor (*F*_[1,56]_ = 12.95; *P* = 0.001), and more evening (*F*_[1,56]_ = 7.77; *P* = 0.007) and morning (*F*_[1,56]_ = 4.11; *P* = 0.047) tension on the Profile of Mood States and lower evening (*F*_[1,38]_ = 35.02; *P* < 0.0001) ratings of mood, less difficulty waking (*F*_[1,35]_ = 14.15; *P* = 0.001) lower physical ailments (*F*_[1,37]_ = 9.42; *P* = 0.004) on the PSI. Cocaine dependent participants reported lower ratings of sleepiness on the evening visual analog scale (*F*_[1,58]_ = 4.54; *P* = 0.037); however, pairwise comparisons were not significant. 

There was a sleep night*group interaction on ratings of restless (*F*_[1,37]_ = 5.72; *P* = 0.022), but pairwise comparisons were not significant. There was also a sleep night*group interaction on ratings of depth (*F*_[1,37]_ = 11.28; *P* = 0.002), with cocaine-dependent participants reporting less depth of sleep after recovery sleep than sleep deprivation (*P* = 0.017) and healthy controls reporting more depth of sleep after recovery sleep than sleep deprivation (*P* = 0.029) on the PSI. There was an effect of age on ratings of depth on the PSI (*F*_[1,37]_ = 6.22; *P* = 0.017) and on evening depression (*F*_[1,56]_ = 5.05; *P* = 0.029), evening vigor (*F*_[1,56]_ = 7.84; *P* = 0.007), and evening (*F*_[1,56]_ = 8.18; *P* = 0.006) and morning (*F*_[1,56]_ = 9.92; *P* = 0.003) tension on the Profile of Mood States.

## 4. Discussion

During an actively prolonged wakeful state following one night of total sleep deprivation, there were no changes in any metabolite in either study group. However, after one recovery sleep night comprised of 8 hours time in bed, *α*-NTP, *β*-NTP, and total NTP significantly increased in cocaine-dependent participants, but not in healthy controls. These results partially fit with previous studies that found no change in the high-energy phosphates or GPC after one night of sleep deprivation [17, 18]. Dorsey et al. [18] did report increases in *β*-NTP, *γ*-NTP, total NTP, and GPC after recovery sleep night in healthy controls. While the current data does reflect an enhancement of NTPs including *β*-NTP, the difference did not reach statistical significance during post hoc comparisons. One possible explanation for this difference is that the current study examined global whole brain  ^[31]^P MRS while Murashita et al. [17] examined only the frontal lobe and Dorsey et al. [18] examined a 5 cm slice of brain centered on the basal ganglia and anterior cingulate. The current results also partially fit with data from a companion study obtained in methadone-maintained participants showing increased *β*-NTP after one recovery sleep night when compared to healthy controls [19]. This indicates that increased energy (ATP) stores after a recovery sleep night are not specific to cocaine-dependent users. Although the elevated levels of *β*-NTP following recovery sleep were common in both methadone-maintained and cocaine dependent participants, the elevated *β*-NTP levels were isolated to the morning following recovery sleep in cocaine-dependent participants, while *β*-NTP elevations were apparent in methadone-maintained participants during active sleep deprivation as well. The occurrence of changes in *β*-NTP observed in methadone-maintained individuals during a state of active prolonged wakefulness following sleep deprivation that continues on into the morning following recovery sleep may be indicative of a greater disruption to sleep homeostatic mechanisms in methadone-maintained versus cocaine dependent individuals. Overall, these data indicate that elevated *β*-NTP following sleep deprivation may reflect a greater susceptibility to the disruptive effect of sleep loss on sleep homeostasis that is common to other forms of substance dependence.

Polysomnography data from both healthy control and cocaine-dependent participants exhibited the typical changes associated with recovery sleep, such as increased sleep efficiency index and slow wave sleep during recovery sleep. While Trksak et al. [19] found that methadone-maintained participants did not exhibit the enhancements of total sleep time and sleep efficiency index normally associated with recovery sleep, in the current study cocaine-dependent subjects did not appear to exhibit disrupted recovery sleep. While the polysomnography results did not reveal statistically significant polysomnography sleep differences in cocaine-dependent participants, the sleep findings are similar to other studies that have reported a mismatch between objective and subjective sleep [2, 18]. In this light, it is interesting that subjective sleep and mood ratings obtained in the current study were worse in cocaine-dependent participants at baseline compared to healthy control participants. 

Overall, cocaine-dependent participants performed significantly worse on the Digit Symbol Substitution Task assessment of psychomotor performance and the Continuous Performance Task assessment of selective attention and/or impulsivity when compared to healthy control participants. These findings are consistent with the majority of previous research demonstrating worse performance in cocaine dependent participants than healthy controls [29], with the largest effects on tests of attention (including the Digit Symbol Substitution Task). Although there are limitations to directly comparing these studies (differences abstinence duration, amount and duration of cocaine use, concurrent use of other substances) these studies consistently find decrements in cognitive performance with cocaine dependence. It was hypothesized that sleep deprivation would decrease cognitive performance in cocaine dependent participants, but sleep deprivation did not significantly affect performance in either group. The findings here fit with its companion study, which also found poorer performance on these same tasks in methadone-maintained participants when compared to healthy controls, which was not worsened by sleep deprivation [30]. These domains of cognitive function may be more resilient to acute sleep loss, but it is possible that the demands of a longer duration period of sleep deprivation could intensify performance decrements in these drug-dependent populations.

A possible limitation to this study is the older age of cocaine dependent participants. A previous study between older and younger cocaine-dependent and older and younger healthy control populations found that older users performed more poorly than controls and younger users on the Digit Span Forward Task, and older users performed more poorly than younger users on the Trail Making Task-A [31]. However, there were no other age*group interactions in that study. To address the age difference between groups in the current study, we included age as a covariate in all of the analyses and found an effect of age only on the evening Digit Symbol Substitution Task, brain expression of PCr, and ratings of depth of sleep, vigor, and tension. A second limitation of the current study may be the limited sample size particularly in the cocaine dependent group. As our primary hypothesis was related to changes in brain bioenergetics following sleep deprivation in cocaine dependent participants, upon interim analysis it became clear that we had obtained robust and significant effects with the current sample size and we ceased data collection. Additionally, a potential limitation of the study may be that MRS imaging was collected from large areas of the brain including temporal, parietal, and frontal cortical regions. In this voxel matrix, there is an inherent bias for occipital and frontal locations, and it includes diencephalic regions such as thalamic and hypothalamic regions, and thus there could be disparate levels of bioenergetics distributed throughout these regions.

## 5. Conclusions 

Collectively, the findings indicate that although changes during active sleep deprivation and following recovery sleep were not identified in cognitive performance data, there were robust changes in brain bioenergetics in cocaine-dependent participants, most profoundly in measures of NTP levels following recovery sleep. One explanation is that the brain increases ATP energy production to serve a compensatory capacity in order to allow the individual to maintain cognitive function and capacity during sleep deprivation. In this context, mechanisms that begin during prolonged wakefulness to assure adequate availability of ATP may fail to downregulate appropriately after recovery sleep in cocaine-dependent individuals. Another explanation may be that sleep deprivation results in a mode of metabolic conservation where energy utilization is reduced to maintain function during prolonged periods of wakefulness. While the functional relevance of changes in brain bioenergetics during and following sleep deprivation has yet to be fully elucidated, these sleep deprivation-induced fluctuations in brain ATP appear to be a prominent aspect of brain changes modulated by sleep homeostatic processes. The enhancements in ATP levels presently observed appear to be at the expense of PCr levels, which are indicative of a change in the creatine kinase equilibrium, potentially indicating that the brain is working harder in a sleep deprived state. The enhanced ATP levels in cocaine-dependent participants in the current study were focused in the morning following recovery sleep, while enhanced brain ATP levels were markedly more pronounced both following recovery sleep and during active sleep deprivation in methadone-maintained participants in Trksak et al. [19]. This may indicate that although both drug-dependent groups exhibit an altered response to sleep deprivation, the impact of acute sleep loss on homeostatic mechanisms may be greater in methadone-maintained individuals than cocaine-dependent individuals. Collectively, these changes in brain ATP levels following recovery sleep in cocaine-dependent participants when compared to healthy control participants are likely indicative of a greater impact of sleep deprivation to sleep homeostatic mechanisms that may reflect an underlying metabolic vulnerability to sleep loss in this population. This may have direct implications for daily function and decision-making and furthermore have an influence on the likelihood of relapse to cocaine use during abstinence. These findings highlight the potential clinical importance of monitoring or improving sleep quality as abstinence progresses [3].

## Figures and Tables

**Table 1:** The top panel shows demographic characteristics and current drug use data of healthy control (HC), cocaine-dependent (COC) participants. Demographics are presented as average ± standard deviation or number of subjects.

	HC	COC
Age yr	34 (3.1)	44.5 (1.8)
Years of cocaine use	—	16 (7.6)
Average weekly cocaine use (days)	—	4.38 (1.9) 6 subjects predominantly smoked cocaine
Average cigarettes per day	—	2 smokers 5.3 (0.4)
Cannabinoid	—	1 subject
Opiates	—	2 subjects

**Table 2:** Polysomnographic assessments of sleep control versus all cocaine-dependent (COC) participants data are displayed as mean (Standard Deviation).

	Sig.	Baseline		Sig.	Recovery		Sig.
		Control	COC		Control	COC	
Sleep onset latency (mins.)		23.3 (6.4)	34.3 (20.4)		10.5 (18.5)	4.6 (2.6)	∗
Waking after sleep onset (mins.)		54.9 (18.5)	66.5 (32.2)		14.7 (2.5)	33.0 (9.3)	∗
Sleep efficiency index		81.8 (4.3)	79.1 (5.2)		94.5 (1.2)	91.8 (1.8)	∗
Total sleep time (hrs.)		6.3 (0.3)	6.3 (0.4)		7.4 (0.2)	7.4 (0.1)	∗
Number of arousals	‡	88.9 (10.1)	54.5 (6.5)		78.2 (8.9)	56.0 (7.2)	
Arousal index		14.5 (2.0)	16.9 (7.8)		10.4 (1.3)	7.1 (1.0)	
Stage 1 (mins.)		49.1 (8.6)	42.5 (3.4)		41.4 (8.0)	30.7 (5.1)	
Stage 2 (mins.)		214.5 (12.7)	238.1 (19.1)		251.8 (11.5)	259.4 (7.8)	∗
Stage 3 (mins.)		27.7 (7.5)	16.7 (7.0)		32.7 (11.9)	18.3 (6.7)	
Stage 4 (mins.)		8.5 (4.4)	7.2 (4.0)		16.9 (6.9)	12.9 (8.5)	
REM (mins.)		79.4 (8.8)	74.3 (8.7)		101.3 (5.7)	121.8 (9.2)	∗
Slow wave sleep (mins.)		132.9 (34.6)	95.7 (36.5)		216.0 (50.6)	143.2 (57.6)	

Significance (Sig.): *sleep night *P* < 0.05; ^‡^group *P* < 0.05.

**Table 3:** Profile of Mood States (POMS), Post-Sleep Inventory (PSI), Stanford Sleepiness Scale (SSS), and Visual Analog Scale (VAS) assessments of sleepiness and mood in control (control) versus cocaine-dependent (COC) participants data are displayed as mean (Standard Deviation).

	Sig.	Baseline		Sig.	Sleep deprived		Sig.	Recovery		Sig.
		Control	COC		Control	COC		Control	COC	
POMS										
Depression PM	‡	3.4 (1.6)	11.6 (4.9)		2.6 (1.4)	3.3 (1.7)		2.5 (1.6)	7.0 (3.5)	
Depression AM		3.4 (1.6)	3.3 (1.3)		3.3 (1.7)	5.0 (2.6)		2.5 (1.8)	4.1 (1.5)	
Vigor PM	‡	16.9 (2.2)	12.0 (2.2)		14.5 (2.2)	10.3 (2.3)		10.1 (2.5)	5.0 (1.5)	∗
Vigor AM		10.5 (2.3)	7.0 (2.0)		7.9 (2.1)	7.1 (1.7)		9.5 (2.3)	4.9 (2.5)	
Fatigue PM		3.4 (0.8)	2.5 (1.2)		3.7 (1.0)	4.0 (1.3)		9.4 (1.7)	13.3 (1.8)	∗
Fatigue AM		4.1 (0.8)	3.5 (0.8)		9.1 (1.8)	9.0 (1.3)	∗	5.8 (1.2)	4.9 (1.2)	
Tension PM	‡	5.3 (1.1)	7.0 (2.4)		5.5 (1.0)	5.9 (1.7)		5.5 (1.2)	8.8 (1.5)	
Tension AM	‡	4.3 (1.0)	4.3 (0.7)		4.7 (1.2)	6.5 (2.0)		4.2 (1.2)	4.3 (0.6)	
PSI										
Restless		6.2 (0.8)	8.1 (1.1)		—	—		8.4 (1.0)	5.3 (1.3)	§
Depth		7.0 (0.8)	7.7 (0.7)		—	—		9.4 (1.0)	4.1 (0.5)	
Mood PM	‡	9.5 (0.7)	5.3 (0.9)		—	—		9.6 (0.7)	4.6 (0.6)	
Mood AM		8.1 (0.7)	5.0 (0.9)		—	—		8.2 (0.8)	5.9 (1.2)	
Rested		6.8 (0.8)	5.7 (1.0)		—	—		5.6 (0.8)	7.6 (1.3)	
Difficulty waking		8.5 (0.9)	3.7 (0.6)		—	—		6.1 (0.8)	3.8 (0.5)	
Physical ailments	‡	8.9 (0.7)	5.3 (0.7)		—	—		8.4 (0.7)	6.0 (0.9)	
Frequency of waking		6.9 (0.6)	5.9 (1.3)		—	—		9.6 (0.9)	5.6 (1.4)	
SSS										
Evening		3.7 (0.4)	4.0 (0.8)		2.6 (0.3)	2.9 (0.7)		5.2 (0.5)	6.0 (0.5)	∗
Morning		3.8 (0.4)	4.1 (0.5)		4.5 (0.5)	5.1 (0.4)		3.9 (0.4)	4.0 (0.7)	
VAS										
Evening		49.4 (6.7)	25.3 (7.8)		57.9 (7.3)	58.9 (7.5)	∗	20.4 (4.4)	14.0 (8.6)	∗
Morning		50.4 (5.9)	41.1 (8.4)		29.0 (6.7)	23.9 (5.4)	∗	41.9 (6.5)	33.6 (6.3)	

Significance (Sig.): *sleep night *P* < 0.05; ^‡^group *P* < 0.05; ^§^sleep night*group interaction *P* < 0.05.
